# Effect of NOS3 gene polymorphism on response to Tricyclic antidepressants in migraine attacks

**Published:** 2014-07-04

**Authors:** Aliasghar Molana, Masoud Mehrpour, Nasim Vousooghi, Mahmoud Reza Hajighasem, Mohammad Taghi Joghataei

**Affiliations:** 1 Department of Neuroscience, School of Advanced Technologies in Medicine, Iran University of Medical Sciences, Tehran, Iran; 2 Department of Neurology, Firoozgar Hospital, Iran University of Medical Sciences, Tehran, Iran; 3 Department of Neuroscience, School of Advanced Technologies in Medicine AND Genetics Laboratory, Iranian National Center for Addiction Studies, Tehran University of Medical Sciences, Tehran, Iran; 4 Department of Neuroscience, School of Advanced Technologies in Medicine AND Brain and Spinal Cord Injury Research Center, Imam Khomeini Hospital, Tehran University of Medical Sciences, Tehran, Iran

**Keywords:** Headache, Nitric Oxide Synthase 3, Migraine, Polymorphism, Tricyclic Antidepressants

## Abstract

**Background: **Migraine is a chronic neurological disorder, characterized by recurrent moderate to severe headaches. Worldwide migraine affects nearly 15%. Studies suggest that genes involved in the production of nitric oxide (NO) may act as genetic factors for migraine. NO synthase 3 (NOS3) by expressing enzyme NOS regulates endothelial derived NO. One class of medications used as first-line treatment in migraine prophylaxis is tricyclic antidepressants (TCAs). The aim of this study was to determine effects of NOS3 gene Glu298Asp polymorphism in the production of NO and response of patients to TCAs in migraine attacks.

**Methods:** A total of 80 migraine patients were invited to participate in the study. Patients recorded the characteristics of their migraine attacks such as frequency of attacks and intensity of headaches for the 1^st^ month of the study. Then peripheral blood samples were taken from all subjects in order to determine patients’ genotype distribution, mRNA expression level of NOS3 and NO content of plasma. Patients were then instructed to use 25 mg nortriptyline at night before bed for 3 months. At the end of 3^rd^ month of the treatment patients again recorded the migraine characteristics for 1 month and blood sampling was performed in order to determine the level of plasma NO.

**Results: **The patients’ genotype distribution for TT, GT, and GG was 9, 24, and 47 subjects, respectively. Mean NO level in patients with TT genotype was less in comparison to GT and GG genotypes before and after use of TCAs (P < 0.05). Mean intensity of headaches in patients with TT genotype was lower in comparison to GT and GG genotypes before and after use of TCAs (based on verbal numerical rating scale). Mean frequency of migraine attacks after use of TCAs was significantly decreased in all genotypes of NOS3 Glu298Asp polymorphism particularly in TT genotype (P < 0.05).

**Conclusion: **Presence of T allele of the Glu298Asp polymorphism may be a factor for TT genotype patients to produce less NO and is a favorable factor for better response to TCAs in reducing migraine attacks in comparison to GT and GG genotypes.

## Introduction

Migraine is a chronic neurological disorder characterized by recurrent moderate to severe headaches. It represents an enormous socioeconomic burden to the individual as well as the society and affects the quality of life.^1^ Typically, the headache is unilateral and pulsating in nature lasting from 2 to 72 h. Associated symptoms may include nausea, vomiting, photophobia, phonophobia, and the pain, which is generally aggravated by physical activity.^2^

Worldwide migraine affects nearly 15% or approximately 1 billion people. It is more common in women at 19% than men at 11%.^3^ Diagnosis of migraine can be made according to the International Headache Society (IHS). The disease most commonly starts between 15 and 24 years of age and occurs most frequently in those who are between 35 and 45 years old.^4^

Migraine is claimed to be a neurovascular disorder.^4^ The intracranial blood vessels are innervated by trigeminal sensory nerve that stores several neuropeptides including substance P and calcitonin gene-related peptide (CGRP).^5^

The generation of migraine occurs in the brain stem. Activation of trigeminovascular system stimulates perivascular trigeminal sensory afferent nerve with the release of vasoactive neuropeptides such as substance P and CGRP and results in neuroinflammation.^6^ High density of CGRP binding sites are present in blood vessels.^7^ G-protein receptor forms the basic receptor protein for CGRP.^8^ CGRP-induced vascular responses are mediated through endothelium pathway. CGRP by binding to its receptors in the endothelium of intracranial blood vessels activates adenyl cyclase thereby increasing cyclic adenosine monophosphate (cAMP) levels. This increase in cAMP levels activates the enzyme nitric oxide synthase (eNOS) which in turn increases the level of nitric oxide (NO). Studies have demonstrated that migraine patients have a higher concentration of plasma NO in comparison to healthy people.^9^ NO which is a potent vasodilator implicated in migraine headaches,^10^ has a strong correlation with CGRP.^11^

NO acts on smooth muscles by activating guanyl cyclase with an ensuring production of cyclic guanosine monophosphate (cGMP). Agents that increase cGMP can activate K-channels. K efflux resulting from K-channel opening causes hyperpolarization, inhibits voltage-gated Ca channels and promotes smooth muscle relaxation.^12^

The information of vasodilatation is conveyed to central neurons in the trigeminal sensory nucleus that in turn relays the pain signals to the higher centers where headache pain is perceived.

Studies suggest that genes involved in the production of NO may act as genetic factors for migraine.^13^ NOS3 by expressing eNOS regulates endothelial derived NO.^14^

The NOS3 gene is located on chromosome 7 and consists of 26 exons.^15^ The NOS3 gene has numerous polymorphisms which among them the Glu298Asp at exon 7 is the only single nucleotide polymorphism (SNP) (guanine to thymine at position 894) which leads to amino acid substitution (from glutamic acid to aspartic acid at position 298).^16^

One of important factors that have been implicated in pathophysiology of migraine is serotonin which plays a role in modulation of pain.^17^ Studies suggest that migraine patients tend to have low levels of serotonin in their brain.^18^ One class of medications which is used as first-line treatment in migraine prophylaxis is TCAs. These agents are effective in preventing migraine, and the response is usually rapid.^19^ TCAs increase the concentration of serotonin in the synaptic cleft by blocking the transport of serotonin back into the presynaptic neurons.^20^ Human trigeminal ganglia express abundant 5HT1B/D receptors and serotonin agonists through 5HT1B/D receptors inhibit trigeminal activity in releasing CGRP and consequently affect the release of NO and migraine attacks.^21^^,^^22^

By considering high prevalence of migraine in society, the aim of this study was to determine the effect of NOS3 gene Glu298Asp polymorphism in the production of NO and investigating the response of this polymorphism to TCAs treatment in migraine attacks.

## Materials and Methods

A total of 80 migraine patients of which 46 were women and 34 were men did not have familial relationships were invited to participate in this study. Patients signed the informed consent prior to entering the study. Patients were matched for age and sex and types of migraine and had a history of migraine for at least 1 year. Age of patients was 35.2 ± 13.6 years. The patients had at least two attacks per month and diagnosed by a specialist based on IHS. The study group consisted of 54 patients suffering from migraine without aura and 26 migraine patients with aura. Patients in the 1^st^ month of study recorded their migraine attack (migraine headache) characteristics such as frequency of migraine attacks and intensity of headaches based on verbal numerical rating scale (VNRS). Then for all subjects peripheral blood sample were collected into sterile tube with ethylenediaminetetraacetic acid in headache free period.

Genomic DNA was isolated from whole blood using QIAamp DNA Blood Mini Kit (Qiagen, Germany). Polymerase chain reaction (PCR) analysis was made with oligonucleotides primers. The sense primer was: 5-CATGAGGCTCAGCCCCAGAAC-3 and the antisense primer was: 5-AGTCAATCCCTTTGGTGCTCAC-3.

The DNA was denatured at 94° C for 1 min and temperature cycling was set at 94° C for 1 min, 63° C for 1 min and 72° C for 1 min for 35 cycles. PCR products were visualized on 2% Agarose gel stained with ethidium bromide. SNP genotyping was performed by Sanger sequencing.

**Figure 1:**
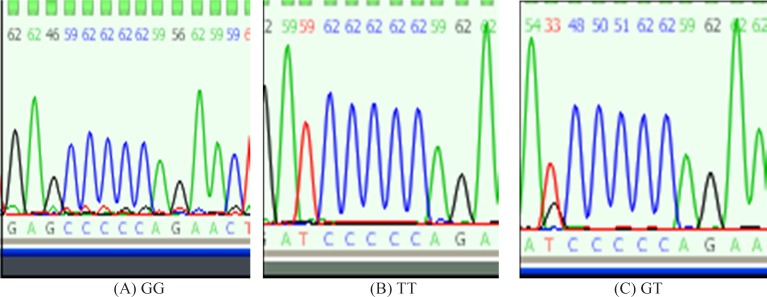
Patients’ genotype determination by use of Sanger sequencing by considering location of the SNP in NOS3: (A) Single black peak indicate homozygote in the form of GG genotype. (B) Single red peak indicate homozygote in the form of TT genotype. (C) Double peak (red and black) indicate heterozygote in the form of GT genotype

Sanger sequencing is a method of DNA sequencing based on the selective incorporation of chain terminating dideoxynucleotides by DNA polymerase during in vitro DNA replication. In this method, dideoxynuycleotides are labeled with fluorescent dye, each of which emit light at different wavelengths which is depicted in the form of a chromatogram which is a diagram of colored peaks that correspond to the nucleotide in that location in the sequence. By investigating the location of the SNP in the gene, genotypes of the patients are identified (Figure 1).

NOS3 m-RNA expression was measured by real-time PCR/reverse transcription-PCR method. In this method, first total RNA was extracted using QIAamp RNA Blood Mini Kit (Qiagen, Germany). Then reverse transcription was performed using 2 µg of total RNA and random hexamers with the following conditions: 65° C for 2 min, 45° C for 60 min, and 95° C for 5 min.

For quantification of mRNA, resulting cDNA was amplified by using specific primers for NOS3 and B-actin. B-actin was used as housekeeping gene. Expression level was quantified as the difference between the number of cycles required to achieve threshold for NOS3 m-RNA and that required for B-actin (delta threshold). The ratio of NOS3 m-RNA expression to B-actin for each sample was calculated. Plasma NO levels in the headache free period was measured. Then, patients used nortriptyline 25 mg tablet at night before bed for 3 month. At the end of 3^rd^ month patients again recorded their frequency of migraine attacks and intensity of headaches for 1 month. Blood samples were again collected in headache free period in order to measure NO level.

In this study, 11 patients stopped using the medication because of the adverse effects of the drug (dry mouth, dizziness, and sedation) and were excluded and subsequently replaced by newly diagnosed patients.

***Statistical analysis***

SPSS for Windows 14.0 (SPSS Inc., Chicago, IL, USA). was used for statistical analysis paired sample t-test and one-way ANOVA was used as statistical methods for comparison. The significance level was set at P < 0.05.

## Results

Patients’ genotype distribution for TT, GT, and GG were 9, 24, and 47 subjects, respectively (T stands for thymine and G for guanine).

**Figure 2:**
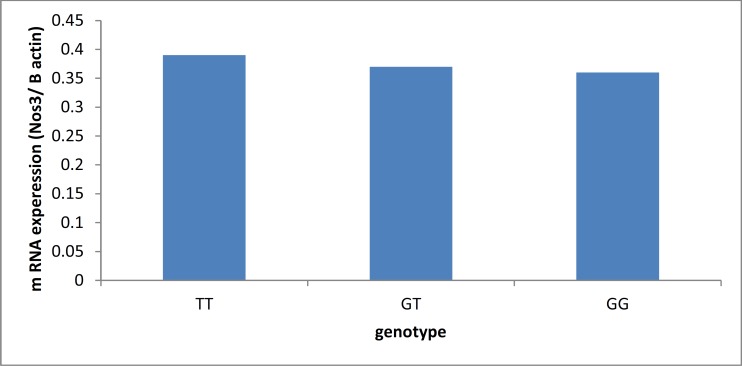
Comparison of the genotypes according to mRNA expression

mRNA expression for genotypes TT, GT, and GG were 0.39 ± 0.11, 0.37 ± 0.06, and 0.36 ± 0.04, respectively (Figure 2).

**Figure 3:**
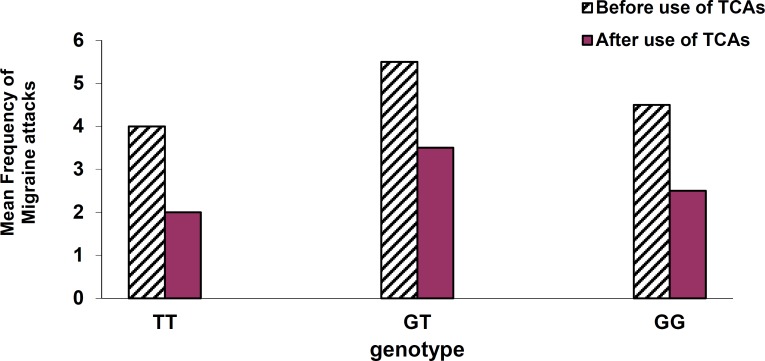
Comparison of the genotypes according to the mean nitric oxide level before and after  use of tricyclic antidepressants

In this study, mean NO level before use of TCAs in patients with genotype TT, GT, and GG were 29.50 ± 0.06 µmol/l, 32.70 ± 0.09 µm/l, and 32.90 ± 0.07 µm/l, respectively. Mean NO level after use of TCAs in patients with genotypes TT, GT, and GG were 28.80 ± 0.05 µm/l, 32.10 ± 0.06 µm/l, and 32.30 ± 0.05 µm, respectively (Figure 3).

**Figure 4:**
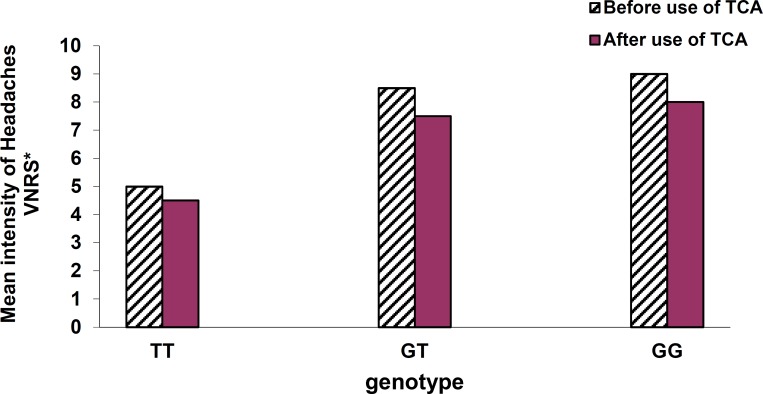
Comparison of the genotypes according to the mean intensity of headaches before and after use of tricyclic antidepressants. Verbal Numerical Rating Scale* (VNRS*): 0 → no pain;  1-3 → mild pain; 4-6 → moderate pain; 7-10 → severe pain

Intensity of headaches based on VNRS before use of TCAs was reported as: medium level in patients with genotype TT (5 ± 1), severe in subjects with GT (8.5 ± 0.5), and severe in patients with GG (9 ± 1). Furthermore, intensity of headache after use of TCAs was reported as: medium level in TT (4.5 ± 0.5), severe level in GT (7.5 ± 0.5), and severe level in GG genotype (8 ± 1) (Figure 4).

**Figure 5:**
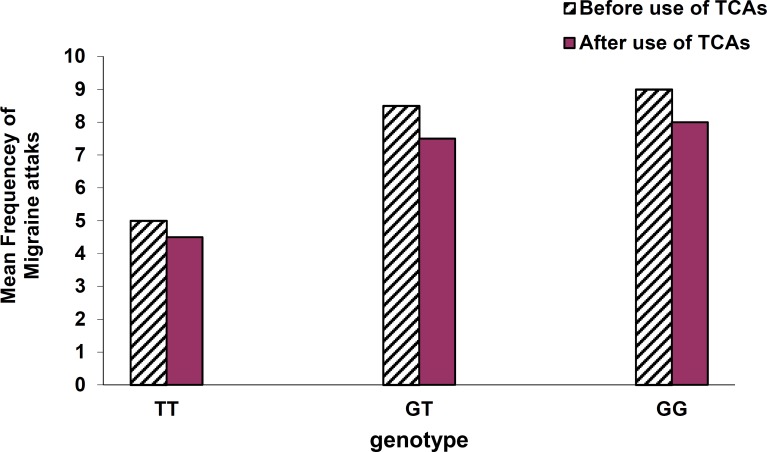
Comparison of the genotypes according to mean frequency of migraine attacks  before and after use of tricyclic antidepressants

Mean frequency of migraine attacks before use of TCAs in patients with genotype TT, GT, and GG was reported as 4 ± 1, 5.5 ± 0.5, and 4.5 ± 0.5, respectively and after use of TCAs the frequencies were reported2 ± 1, 2.5 ± 0.5, and 2 ± 1, respectively (Figure 5).

## Discussion

Studies have shown that the NOS3 Glu298Asp variation is fairly widespread in human population, with 35-40% prevalence of the T allele in Caucasians and less prevalence in African, Americans, and Asians.^23^

In this study, the percentage of TT, GT, and GG genotypes were 11.2%, 30%, and 58.8%, respectively. And overall prevalence of T allele was 26.2%.

There was no significant correlation between the polymorphism and age, sex, and type of migraine patients.

NOS3 mRNA expression tended to be higher in TT genotype patients in comparison with GT and GG genotypes, but based on one-way ANOVA test and with P < 0.05 the difference was not statistically significant. Mean NO level produced by NOS3 genotypes after use of TCAs based on paired sample t-test with P < 0.05 did not change significantly. Furthermore, mean NO level production before and after use of TCAs by using ANOVA test with P < 0.05 in patients with TT genotype were less in comparison with GT and GG genotypes. This finding indicates that NOS3 variant expression leads to conformational changes in eNOS protein and reduced activity of the enzyme.^24^

Mean intensity of headaches based on VNRS before and after use of TCAs in patients with TT genotype was reported lower in comparison with GT and GG genotypes. This finding may show a direct correlation between NO level and intensity of headaches.

Mean frequency of migraine attacks after use of TCAs based on paired sample t-test with P < 0.05 was significantly decreased in all genotypes of NOS3. Mean percentage reduction in attacks in TT, GT, and GG genotypes were 50%, 45.5%, and 44.6%, respectively. This result by use of ANOVA test with P < 0.05 suggests that the presence of T allele of the Glu298Asp polymorphism may be a favorable factor in responding to TCAs in reducing migraine attacks.

## Conclusion

In this study, homozygote variant (TT genotype) of NOS3 gene Glu298Asp polymorphism produced less NO level in comparison with GT and GG genotypes. Use of TCAs had no significant effect in intensity of headache in migraine patients, but by decreasing frequency of migraine attacks had an inhibitory role in migraine generation, particularly in patients with TT genotype.

## Notes

**How to cite this article: **Molana A, Mehrpour M, Vousooghi N, Hajighasem MR, Joghataei MT. Effect of NOS3 gene polymorphism on response to Tricyclic antidepressants in migraine attacks. Iran J Neurol 2014; 13(3): 154-9.
